# The Diagnostic Value of DNA Methylation in Leukemia: A Systematic Review and Meta-Analysis

**DOI:** 10.1371/journal.pone.0096822

**Published:** 2014-05-08

**Authors:** Danjie Jiang, Qingxiao Hong, Yusheng Shen, Yan Xu, Huangkai Zhu, Yirun Li, Chunjing Xu, Guifang Ouyang, Shiwei Duan

**Affiliations:** 1 Zhejiang Provincial Key Laboratory of Pathophysiology, School of Medicine, Ningbo University, Ningbo, Zhejiang, China; 2 Department of Hematology, Ningbo First Hospital, Ningbo, Zhejiang, China; University of Bonn, Institut of experimental hematology and transfusion medicine, Germany

## Abstract

**Background:**

Accumulating evidence supports a role of DNA methylation in the pathogenesis of leukemia. The aim of our study was to evaluate the potential genes with aberrant DNA methylation in the prediction of leukemia risk by a comprehensive meta-analysis of the published data.

**Methods:**

A series of meta-analyses were done among the eligible studies that were harvested after a careful filtration of the searching results from PubMed literature database. Mantel-Haenszel odds ratios and 95% confidence intervals were computed for each methylation event assuming the appropriate model.

**Results:**

A total of 535 publications were initially retrieved from PubMed literature database. After a three-step filtration, we harvested 41 case-control articles that studied the role of gene methylation in the prediction of leukemia risk. Among the involving 30 genes, 20 genes were shown to be aberrantly methylated in the leukemia patients. A further subgroup meta-analysis by subtype of leukemia showed that *CDKN2A, CDKN2B, ID4* genes were significantly hypermethylated in acute myeloid leukemia.

**Conclusions:**

Our meta-analyses identified strong associations between a number of genes with aberrant DNA methylation and leukemia. Further studies should be required to confirm the results in the future.

## Introduction

Leukemia is a common malignant disease of hematopoietic system, caused by unbalanced hematopoietic cells proliferation and death [1]. The development and progression of leukemia is complex. Based on the speed of disease progression and the types of affected white blood cell, leukemia can be divided into four most common types of leukemia, which comprise acute myeloid leukemia (AML), chronic myeloid leukemia (CML), acute lymphocytic leukemia (ALL) and chronic lymphocytic leukemia (CLL) (http://www.nlm.nih.gov/medlineplus/leukemia.html).

Although tremendous efforts have been made in the identification of susceptible factors of leukemia [2], [3], the pathogenesis of leukemia is not fully clarified [4]. Environmental factors, such as high benzene exposure, radiation, electrical work, are shown to be associated with the development of leukemia [5], [6]. Meanwhile, leukemia is known to be associated with the accumulation of defects in a wide range of cancer genes [7].

Many genetic and epigenetic alternations were found to play an important role in leukemia pathogenesis [8], [9]. Previous study has indicated aberrant DNA methylation associated with leukemogenesis [10]. As a typical epigenetic modifications, aberrant DNA methylation was observed in lymphoid/hematopoietic malignancies, including AML [4], [11], CML [12], [13], ALL [1], [14], and CLL [15], [16].

These aberrant patterns of DNA methylation in leukemia can be useful for cancer risk prediction. Recent advances attest to the great promise of DNA methylation markers as powerful tools in the clinic [17]–[19]. Meta-analysis can generate a more objective evaluation of candidate genes DNA methylation and the risks of leukemia, based on the conclusions of uncertainty and disagreements. Here we perform comprehensive meta-analyses based on the accumulating leukemia association studies on DNA methylation to better identify biomarkers with aberrant DNA methylation in leukemia. The goal of our study was to summarize the genes with aberrant DNA methylation as promising biomarkers for leukemia risk prediction.

## Materials and Methods

### Search Strategy

**Figure 1:**
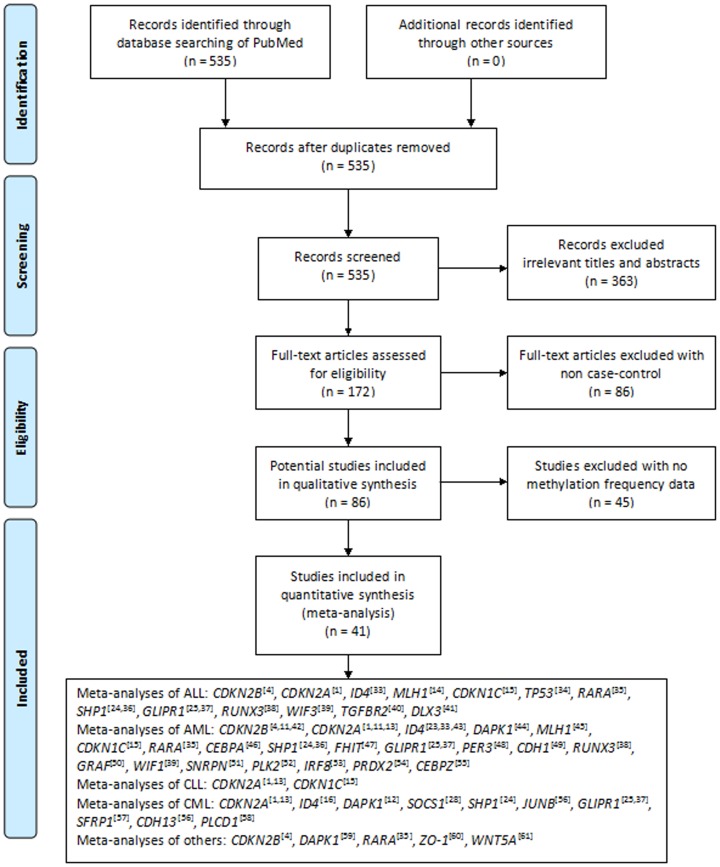
Flow diagram of the stepwise selection from relevant studies.

A systematic literature search was performed in PubMed by using “leukemia” and “DNA methylation” as the search terms for articles updated until December 25, 2013. The search was limited to articles published in English and Chinese. Articles in the search output were surveyed according to their titles and abstracts. Studies were selected if they met the following criteria: 1) they were case-control associations of gene methylation with the risk of leukemia in humans; 2) they had sufficient methylation information to calculate the odd ratios (ORs) and 95% confidential intervals (CIs) for the meta-analysis. The selection procedures of studies were illustrated in the flow chart of Figure 1.

### Data Extraction

For the eligible articles, we extracted the following information: first author’s name, published year, PubMed ID, disease category (AML, ALL, CML, CLL or others), the numbers of cases and controls. All the data were extracted by four authors (DJ, YX, HZ and CX). A consensus was reached through a rigorous discussion when there existed conflicting evaluations.

### Meta-analysis

Review manager 5 was used for meta-analysis. Mantel-Haenszel ORs and their 95% CIs were computed for each gene to evaluate the contribution of gene methylation to the risk of leukemia. Heterogeneity of the studies in the meta-analysis was evaluated by I^2^ metric [20], [21]. A random-effect model was used when there existed heterogeneity in the meta-analysis (I^2^>50%), otherwise a fixed-effect model was applied for the meta-analysis [22].

## Results

### Study Characteristics

A total of 535 studies were retrieved from the PubMed literature database after searching the keywords of “leukemia” and “DNA methylation”. After a series of selection procedure shown in Figure 1, we excluded 363 irrelevant studies, 86 non-case-control studies, and 45 studies without methylation data. Finally, 41 case-control studies were qualified for our meta-analyses. These comprised 15 ALL studies, 31 AML studies, 4 CLL studies, 13 CML studies, and 5 other studies (including 1 on acute promyelocytic leukemia, and 4 on undefined leukemia). The 41 case-control studies were involved with 30 genes among 1640 healthy individuals and 2587 leukemia patients. Among the tested genes, there were 19 genes with only one report, 3 genes with 2 reports, and 8 genes with 3 or more reports (Table 1).

**Table 1:** Genes differently methylated in case-control studies from leukemia subjects.

Gene	Studies	Overall OR (95% CI)^a^	P value
*CDKN2A*	12	3.53 [1.43, 8.73]	<0.01
*GLIPR1*	6	5.96 [2.29, 15.46]	<0.01
*CDKN2B*	5	9.67 [2.48, 37.75]	<0.01
*SHP1*	5	29.27 [6.80, 125.99]	<0.01
*ID4*	5	45.24 [11.02, 185.78]	<0.01
*DAPK1*	3	28.85 [5.54, 150.14]	<0.01
*CDKN1C*	3	6.16 [0.66, 57.72]	0.11
*RARA*	3	3.46 [0.65, 18.39]	0.15
*RUNX3*	2	11.91 [1.45, 97.86]	0.02
*WIF1*	2	14.15 [1.78, 112.81]	0.01
*MLH1*	2	5.93 [0.27, 130.34]	0.26
*SOCS1*	1	0.10 [0.01, 0.78]	0.03
*JUNB*	1	55.00 [1.86, 1622.60]	0.02
*ZO-1*	1	45.00 [2.01, 1006.75]	0.02
*WNT5A*	1	121.51 [7.08, 2085.83]	<0.01
*PER3*	1	104.27 [6.33, 1718.74]	<0.01
*CDH1*	1	33.00 [1.78, 610.61]	0.02
*GRAF*	1	81.54 [4.82, 1379.56]	<0.01
*SNRPN*	1	25.00 [1.39, 449.48]	0.03
*PLK2*	1	67.34 [3.77, 1204.54]	<0.01
*PRDX2*	1	22.32 [1.32, 377.72]	0.03
*PLCD1*	1	39.38 [2.21, 702.41]	0.01
*CEBPZ*	1	35.84 [2.12, 604.80]	0.01
*TP53*	1	3.40 [0.16, 73.57]	0.44
*CEBPA*	1	2.53 [0.14, 47.18]	0.53
*FHIT*	1	4.80 [0.27, 85.35]	0.29
*SFRP1*	1	1.54 [0.17, 14.09]	0.70
*CDH13*	1	11.00 [0.46, 263.53]	0.14
*DLX3*	1	1.66 [0.48, 5.65]	0.42
*IRF8*	1	6.41 [0.34, 120.24]	0.21

a Odds ratio (OR) describes the likelihood of gene methylation observed in leukemia cases compared to controls.

### Meta-analysis of the Association Studies between Gene and the Risk of Leukemia

**Figure 2:**
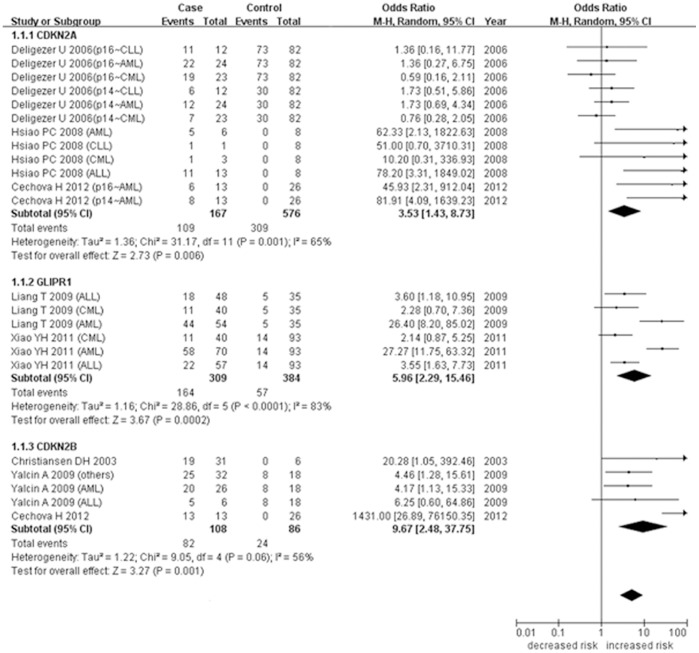
Correlation between *CDKN2A/GLIPR1/CDKN2B* methylation and leukemia in the meta-analysis.

As shown in Figure 2, the meta-analysis of *CDKN2A* methylation was involved with 12 case-control studies among 576 controls and 167 cases. Our results showed a significant heterogeneity among the 12 studies (I^2^ = 65%). In addition, the meta-analysis indicated that hypermethylation of *CDKN2A* gene was associated with the increased risk of leukemia (P = 0.006, OR = 3.53, 95% CI = 1.43–8.73). Meta-analysis between *GLIPR1* methylation and leukemia was involved with 6 case-control studies among 384 controls and 309 cases (Figure 2). The meta-analysis showed a significant heterogeneity (I^2^ = 83%) among these studies, and revealed that *GLIPR1* hypermethylation was associated with the increased risk of leukemia (P = 0.0002, OR = 5.96, 95% CI = 2.29–15.46). Meta-analysis of *CDKN2B* methylation with leukemia was carried out in 5 studies among 86 controls and 108 cases (Figure 2). Our results showed a significant heterogeneity among the 5 case-control studies (I^2^ = 56%), and revealed that hypermethylation of *CDKN2B* gene was associated with the increased risk of leukemia (P = 0.001, OR = 9.67, 95% CI = 2.48–37.75).

Meta-analysis of *SHP1* methylation was involved with 5 case-control studies among 46 controls and 104 cases. Our results indicated that hypermethylation of SHP1 gene was associated with the increased risk of leukemia (P<0.00001, OR = 29.27, 95% CI = 6.80–125.99). Meta-analysis between *ID4* methylation and leukemia was involved with 5 case-control studies among 78 controls and 165 cases. The results revealed that *ID4* hypermethylation was associated with the increased risk of leukemia (P<0.00001, OR = 45.24, 95% CI = 11.02–185.78). Meta-analysis of *DAPK1* methylation with leukemia was carried out in 3 studies among 29 controls and 169 cases. Our results showed that hypermethylation of *DAPK1* gene was associated with the increased risk of leukemia (P<0.0001, OR = 28.85, 95% CI = 5.54–150.14). In addition, our results were unable to observe significant association of the methylation of *CDKN1C* and *RARA* genes with leukemia.

### Meta-analysis of the Association Studies between Gene and the Risk of AML

**Figure 3:**
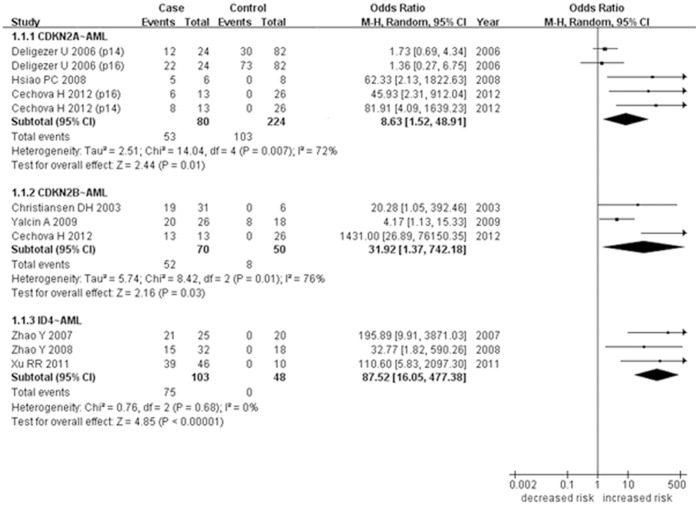
Correlation between *CDKN2A/CDKN2B/ID4* methylation and AML in the meta-analysis.

We further performed a breakdown meta-analysis by leukemia subtypes in the above-mentioned studies (Table 2). As shown in Figure 3, meta-analysis of *CDKN2A* methylation in AML was involved with 5 case-control studies among 80 cases and 224 controls. A significant heterogeneity was observed among the 5 studies (I^2^ = 72%). Our results showed that *CDKN2A* hypermethylation was associated with the increased risk of AML (P = 0.01, OR = 8.63, 95% CI = 1.52–48.91). The meta-analysis between *CDKN2B* methylation and AML was involved with 3 studies among 70 cases and 50 controls (Figure 3). There existed significant heterogeneity among the 3 studies in the meta-analysis of *CDKN2B* methylation (I^2^ = 76%). Our results showed *CDKN2B* hypermethylation was associated with the increased risk of AML (P = 0.03, OR = 31.92, 95% CI = 1.37–742.18). As shown in Figure 3, the meta-analysis of *ID4* methylation was involved with 3 studies among 103 cases and 48 controls. The results showed that *ID4* hypermethylation was associated with the increased risk of AML (P<0.00001, OR = 87.52, 95% CI = 16.05–477.38).

**Table 2:** Genes differently methylated in case-control studies from different kinds of leukemia subjects.

Gene∼Disease	Studies	Overall OR (95% CI)^a^	P value
*CDKN2A*∼AML	5	8.63 [1.52, 48.91]	0.01
*CDKN2B*∼AML	3	31.92 [1.37, 742.18]	0.03
*CDKN2A*∼CML	3	0.82 [0.38, 1.74]	0.6
*CDKN2A∼CLL*	3	2.04 [0.74, 5.59]	0.17
*ID4*∼AML	3	87.52 [16.05, 477.38]	<0.01

a Odds ratio (OR) describes the likelihood of gene methylation observed in leukemia cases compared to controls.

## Discussion

Numerous studies have found that DNA methylation of different genes was associated with the risk of leukemia [11]–[13], [23]–[25], which implicated a potential role of DNA methylation in the prediction and prognostication for leukemia.

De novo methylation of the 5′CpG island has been reported as an alternative mechanism of inactivation for tumor suppressor genes *CDKN2A* and *CDKN2B* [26]. De novo methylation of *CDKN2B* and *CDKN2A* CpG islands is frequent in malignant transformation [11]. According to the results of our meta-analysis, the aberrant DNA methylation at *CDKN2A* gene and *CDKN2B* gene were risk factors for leukemia, especially for AML. In the subgroup analysis, we found that DNA methylation of *CDKN2A* gene was significantly associated with AML, but not with CML or CLL. A microarray analysis in 2011 identified that glioma pathogenesis-related protein 1 (*GLIPR1*) was a methylation-silenced gene in the AML patients, and might serve as a marker to monitor the therapeutic effect of AML [25]. Our analysis also demonstrated that DNA methylation of *GLIPR1* gene was a risk factor for leukemia. *GLIPR1* is a pleiotropic protein involved in cell proliferation, tumor growth and apoptosis, and it may affect G protein signaling and cell cycle regulation [27]. *SOCS1* is an important protein in the JAK/STAT pathway, and plays a key role in the downstream regulation of BCR-ABL protein kinase [28]. Our results showed that DNA methylation of *SOCS1* gene was a protective factor for leukemia. *SHP1* is tumor suppressor gene involved in the regulation of cell cycle control and apoptosis [29]. *SHP1* is negative regulator of the Jak/STAT signaling pathway that is implicated in leukemogenesis. Promoter methylation of *SHP1* gene is able to silence its gene expression, and was frequently detected in various kinds of leukemias and lymphoma [24]. Our results showed that aberrant DNA methylation of *SHP1* gene was a risk factor for leukemia. Inhibitor of DNA binding protein 4 (*ID4*) is a member of the dominant-negative basic helix-loop-helix transcription factor family that lacks DNA binding activity and has tumor suppressor function. Promoter of *ID4* is consistently methylated to various degrees in CLL cells, and increased promoter methylation in a univariable analysis was shown to be correlated with shortened patient survival [30]. In our results of analysis, the aberrant DNA methylation at *ID4* gene was a risk factor for leukemia, especially for AML. Death-associated protein kinase 1 (*DAPK1*), a tumor suppressor, is a rate-limiting effecter in an endoplasmic reticulum stress-dependent apoptotic pathway [31]. Aberrant DNA methylation and concomitant transcriptional silencing of *DAPK1* have been demonstrated to be key pathogenic events in CLL [32]. Our study identified the aberrant DNA methylation at *DAPK1* gene was a risk factor for leukemia.

The current meta-analysis has some limitations. Firstly, selection bias is inevitable due to the search strategy restricted to articles published in English or Chinese. Secondly, some large between-study heterogeneity existed in our meta-analyses. This phenomenon may be caused by the facts that different subtypes of leukemia were not separated in the first place due to the limited studies, and that different regions of the same gene were tested for methylation in the involved studies. Thirdly, this analysis was performed at the study level, which limited ability to explore the potential for confounding by various demographic and clinical factors (e.g. ethnicity, hormone, different treatments). Fourthly, most of the studies we selected were performed with Methylation-Specific PCR (MSP) and the status of DNA methylation was qualitative (M+ or M−), and it also limited the scope of our analysis. Finally, the efficiency and accuracy of statistical analysis may be influenced by the moderate amount of subjects. We expect more samples being tested in the future to draw a more reliable conclusion.

In conclusion, the results of this study indicated that certain genes DNA methylation was independently associated with the risk of leukemia, especially some kinds of leukemia. Also more studies should be required to confirm the results in the future. DNA methylation has a very strong potential to be a useful biomarker for predicting, prognostication and prediction of response to chemotherapy of leukemia.

## Supporting Information

Checklist S1 **PRISMA Checklist.**
(DOC)
