# Supplementary material for: The Diagnostic Value of DNA Methylation in Leukemia: A Systematic Review and Meta-Analysis
Source: PLoS One. 2014 May 8;9(5):e96822. doi: 10.1371/journal.pone.0096822 (PMC4014555; doi:10.1371/journal.pone.0096822)
Supplement: Checklist S1 — PRISMA Checklist. (DOC) [file pone.0096822.s001.doc]

| **Section/topic** | **#** | **Checklist item** | **Reported on page #** |
| --- | --- | --- | --- |
| **TITLE** | | |  |
| Title | 1 | The diagnostic value of DNA methylation in leukemia: a systematic review and meta-analysis | Title |
| **ABSTRACT** | | |  |
| Structured summary | 2 | Background: Accumulating evidence supports a role of DNA methylation in the pathogenesis of leukemia. The aim of our study was to evaluate the potential genes with aberrant DNA methylation in the prediction of leukemia risk by a comprehensive meta-analysis of the published data.  Methods: A series of meta-analyses were done among the eligible studies that were harvested after a careful filtration of the searching results from PubMed literature database. Mantel-Haenszel odds ratios and 95% confidence intervals were computed for each methylation event assuming the appropriate model.  Results: A total of 535 publications were initially retrieved from PubMed literature database. After a three-step filtration, we harvested 41 case-control articles that studied the role of gene methylation in the prediction of leukemia risk. Among the involving 30 genes, 20 genes were shown to be aberrantly methylated in the leukemia patients. A further subgroup meta-analysis by subtype of leukemia showed that CDKN2A, CDKN2B, ID4 genes were significantly hypermethylated in acute myeloid leukemia.  Conclusions: Our meta-analyses identified strong associations between a number of genes with aberrant DNA methylation and leukemia. Further studies should be required to confirm the results in the future. | Abstract |
| **INTRODUCTION** | | |  |
| Rationale | 3 | Leukemia is a common malignant disease of hematopoietic system, caused by unbalanced hematopoietic cells proliferation and death. The development and progression of leukemia is complex. Based on the speed of disease progression and the types of affected white blood cell, leukemia can be divided into four most common types of leukemia, which comprise acute myeloid leukemia (AML), chronic myeloid leukemia (CML), acute lymphocytic leukemia (ALL) and chronic lymphocytic leukemia (CLL) (http://www.nlm.nih.gov/medlineplus/leukemia.html).  Although tremendous efforts have been made in the identification of susceptible factors of leukemia, the pathogenesis of leukemia is not fully clarified. Environmental factors, such as high benzene exposure, radiation, electrical work, are shown to be associated with the development of leukemia. Meanwhile, leukemia is known to be associated with the accumulation of defects in a wide range of cancer genes.  Many genetic and epigenetic alternations were found to play an important role in leukemia pathogenesis. Previous study has indicated aberrant DNA methylation associated with leukemogenesis. As a typical epigenetic modifications, aberrant DNA methylation was observed in lymphoid/hematopoietic malignancies, including AML, CML, ALL, and CLL. | Introduction |
| Objectives | 4 | These aberrant patterns of DNA methylation in leukemia can be useful for cancer risk prediction. Recent advances attest to the great promise of DNA methylation markers as powerful tools in the clinic. Meta-analysis can generate a more objective evaluation of candidate genes DNA methylation and the risks of leukemia, based on the conclusions of uncertainty and disagreements. Here we perform comprehensive meta-analyses based on the accumulating leukemia association studies on DNA methylation to better identify biomarkers with aberrant DNA methylation in leukemia. The goal of our study was to summarize the genes with aberrant DNA methylation as promising biomarkers for leukemia risk prediction. | Introduction |
| **METHODS** | | |  |
| Protocol and registration | 5 | No. |  |
| Eligibility criteria | 6 | Studies were selected if they met the following criteria: 1) they were case-control associations of gene methylation with the risk of leukemia in humans; 2) they had sufficient methylation information to calculate the odd ratios (ORs) and 95% confidential intervals (CIs) for the meta-analysis. | Materials and Methods |
| Information sources | 7 | Articles updated until December 25, 2013 in PubMed. | Materials and Methods |
| Search | 8 | A systematic literature search was performed in PubMed by using "leukemia" and "DNA methylation" as the search terms. The search was limited to articles published in English and Chinese. | Materials and Methods |
| Study selection | 9 | A total of 535 studies were retrieved from the PubMed literature database after searching the keywords of "leukemia" and "DNA methylation". After a series of selection procedure, we excluded 363 irrelevant studies, 86 non-case-control studies, and 45 studies without methylation data. Finally, 41 case-control studies were qualified for our meta-analyses. These comprised 15 ALL studies, 31 AML studies, 4 CLL studies, 13 CML studies, and 5 other studies (including 1 on acute promyelocytic leukemia, and 4 on undefined leukemia). | Materials and Methods |
| Data collection process | 10 | For the eligible articles, we extracted the following information: first author's name, published year, PubMed ID, disease category (AML, ALL, CML, CLL or others), the numbers of cases and controls. All the data were extracted by four authors (DJ, YX, HZ and CX). A consensus was reached through a rigorous discussion when there existed conflicting evaluations. | Materials and Methods |
| Data items | 11 | The 43 case-control studies were involved with 30 genes among 1640 healthy individuals and 2587 leukemia patients. Among the tested genes, there were 19 genes with only one report, 3 genes with 2 reports, and 8 genes with 3 or more reports. | Materials and Methods |
| Risk of bias in individual studies | 12 | Heterogeneity of the studies in the meta-analysis was evaluated by I^2^ metric . A random-effect model was used when there existed heterogeneity in the meta-analysis (I^2^ > 50%), otherwise a fixed-effect model was applied for the meta-analysis. | Materials and Methods |
| Summary measures | 13 | Review manager 5 was used for meta-analysis. Mantel-Haenszel ORs and their 95% CIs were computed for each gene to evaluate the contribution of gene methylation to the risk of leukemia. | Materials and Methods |
| Synthesis of results | 14 | For the 30 genes in 43 studies, we evaluated overall ORs in case-control studies from leukemia subjects. | Materials and Methods |

Page 1 of 2

| **Section/topic** | **#** | **Checklist item** | **Reported on page #** |
| --- | --- | --- | --- |
| Risk of bias across studies | 15 | Some gene DNA methylation only covered limited studies and we did not separate the different kinds of diseases in the first place, the results maybe have no vigorous power. | Materials and Methods |
| Additional analyses | 16 | No. |  |
| **RESULTS** | | |  |
| Study selection | 17 | A total of 535 studies were retrieved from the PubMed literature database after searching the keywords of "leukemia" and "DNA methylation". After a series of selection procedure, we excluded 363 irrelevant studies, 86 non-case-control studies, and 45 studies without methylation data. Finally, 41 case-control studies were qualified for our meta-analyses. These comprised 15 ALL studies, 31 AML studies, 4 CLL studies, 13 CML studies, and 5 other studies (including 1 on acute promyelocytic leukemia, and 4 on undefined leukemia). The process of study selection was in Figure 1. | Results |
| Study characteristics | 18 | A total of 535 studies were retrieved from the PubMed literature database after searching the keywords of "leukemia" and "DNA methylation". After a series of selection procedure shown in Figure 1, we excluded 363 irrelevant studies, 86 non-case-control studies, and 45 studies without methylation data. Finally, 41 case-control studies were qualified for our meta-analyses. These comprised 15 ALL studies, 31 AML studies, 4 CLL studies, 13 CML studies, and 5 other studies (including 1 on acute promyelocytic leukemia, and 4 on undefined leukemia). The 41 case-control studies were involved with 30 genes among 1640 healthy individuals and 2587 leukemia patients. Among the tested genes, there were 19 genes with only one report, 3 genes with 2 reports, and 8 genes with 3 or more reports (Table 1). | Results |
| Risk of bias within studies | 19 | The results of tests of heterogeneity are in Figure 2 and Figure 3. | Results |
| Results of individual studies | 20 | The results of individual studies are in Table 1 and Table 2. | Results |
| Synthesis of results | 21 | The results are in Table 1 and Table 2. | Results |
| Risk of bias across studies | 22 | Some gene DNA methylation only covered limited studies and we did not separate the different kinds of diseases in the first place, the results maybe have no vigorous power. | Results |
| Additional analysis | 23 | No. |  |
| **DISCUSSION** | | |  |
| Summary of evidence | 24 | Numerous studies have found that DNA methylation of different genes was associated with the risk of leukemia, which implicated a potential role of DNA methylation in the prediction and prognostication for leukemia.  De novo methylation of the 5’CpG island has been reported as an alternative mechanism of inactivation for tumor suppressor genes CDKN2A and CDKN2B. De novo methylation of CDKN2B and CDKN2A CpG islands is frequent in malignant transformation. According to the results of our meta-analysis, the aberrant DNA methylation at CDKN2A gene and CDKN2B gene were risk factors for leukemia, especially for AML. In the subgroup analysis, we found that DNA methylation of CDKN2A gene was significantly associated with AML, but not with CML or CLL. A microarray analysis in 2011 identified that glioma pathogenesis‑related protein 1 (GLIPR1) was a methylation-silenced gene in the AML patients, and might serve as a marker to monitor the therapeutic effect of AML. Our analysis also demonstrated that DNA methylation of GLIPR1 gene was a risk factor for leukemia. GLIPR1 is a pleiotropic protein involved in cell proliferation, tumor growth and apoptosis, and it may affect G protein signaling and cell cycle regulation. SOCS1 is an important protein in the JAK/STAT pathway, and plays a key role in the downstream regulation of BCR-ABL protein kinase. Our results showed that DNA methylation of SOCS1 gene was a protective factor for leukemia. SHP1 is tumor suppressor gene involved in the regulation of cell cycle control and apoptosis. SHP1 is negative regulator of the Jak/STAT signaling pathway that is implicated in leukemogenesis. Promoter methylation of SHP1 gene is able to silence its gene expression, and was frequently detected in various kinds of leukemias and lymphoma. Our results showed that aberrant DNA methylation of SHP1 gene was a risk factor for leukemia. Inhibitor of DNA binding protein 4 (ID4) is a member of the dominant-negative basic helix-loop-helix transcription factor family that lacks DNA binding activity and has tumor suppressor function. Promoter of ID4 is consistently methylated to various degrees in CLL cells, and increased promoter methylation in a univariable analysis was shown to be correlated with shortened patient survival. In our results of analysis, the aberrant DNA methylation at ID4 gene was a risk factor for leukemia, especially for AML. Death-associated protein kinase 1 (DAPK1), a tumor suppressor, is a rate-limiting effecter in an endoplasmic reticulum stress-dependent apoptotic pathway. Aberrant DNA methylation and concomitant transcriptional silencing of DAPK1 have been demonstrated to be key pathogenic events in CLL [32]. Our study identified the aberrant DNA methylation at DAPK1 gene was a risk factor for leukemia. | Discussion |
| Limitations | 25 | The current meta-analysis has some limitations. Firstly, selection bias is inevitable due to the search strategy restricted to articles published in English or Chinese. Secondly, some gene DNA methylation only covered limited studies and we did not separate the different kinds of diseases in the first place, the results maybe have no vigorous power. Thirdly, this analysis was performed at the study level, which limited ability to explore the potential for confounding by various demographic and clinical factors (e.g. ethnicity, hormone, different treatments). Fourthly, most of the studies we selected were performed with Methylation-Specific PCR (MSP) and the status of DNA methylation was qualitative (M+ or M-), and it also limited the scope of our analysis. | Discussion |
| Conclusions | 26 | In conclusion, the results of this study indicated that certain genes DNA methylation was independently associated with the risk of leukemia, especially some kinds of leukemia. Also more studies should be required to confirm the results in the future. DNA methylation has a very strong potential to be a useful biomarker for predicting, prognostication and prediction of response to chemotherapy of leukemia. | Discussion |
| **FUNDING** | | |  |
| Funding | 27 | The research was supported by the grants from National Natural Science Foundation of China (31100919 and 81371469), Natural Science Foundation of Zhejiang Province (LR13H020003), K. C. Wong Magna Fund in Ningbo University, and Ningbo Social Development Research Projects (2010C50019 and 2012C50032). |  |

*From:*  Moher D, Liberati A, Tetzlaff J, Altman DG, The PRISMA Group (2009). Preferred Reporting Items for Systematic Reviews and Meta-Analyses: The PRISMA Statement. PLoS Med 6(6): e1000097. doi:10.1371/journal.pmed1000097

For more information, visit: **www.prisma-statement.org**.

Page 2 of 2
